# Centralized nucleation in online networks leads to high social inequality

**DOI:** 10.1007/s41109-018-0102-3

**Published:** 2018-10-04

**Authors:** Yaniv Dover, Guy Kelman

**Affiliations:** The Hebrew University of Jerusalem, Jerusalem, Israel

## Abstract

Even though the heterogeneity of centrality in social networks is well documented, its role and effect on network stability in real life remains unclear. The literature roughly suggests that network structure is such that networks have an “inner” highly-connected nucleus and, in contrast, sparse outer shells. But to what extent is the existence of this nucleus crucial for the survival of a network? To what extent is the outer shells’ much larger population essential to the longevity of the network? Furthermore, as a network grows and forms, theoretically speaking, network structure should be dependent on the patterns of change of degree centrality, i.e., social mobility between centrality shells. What is the role of social mobility in the formation of the nucleus-to-periphery profile, and is it related to network lifetime? Here, we explore these questions using data collected covering over a decade of activity from more than 10, 000 networked communities, with more than 134,000 users. We find that: **(i)** social mobility is, on average, negative but that, **(ii)** the higher the social mobility of the members of the network, the more stable and long-living the network is. Further, **(iii)** the network is, indeed, composed of two phases - a large but ephemeral sparsely connected “cloud” of actors, that nucleates around a *highly stable* nucleus of users. Lastly, (iv) networked communities which maintain a specific nucleus-to-periphery ratio *η*, i.e., a ratio of the size of the nucleus to periphery of around $\eta =\frac{1}{4}$, have a greater chance of survival. We find that deviations from this nucleus-to-periphery ratio predict a collapse of network activity, especially in the case of younger communities.

**Electronic supplementary material:**

The online version of this article (10.1007/s41109-018-0102-3) contains supplementary material, which is available to authorized users.

## Introduction

Previous studies show that social network degree centrality^1^ is highly heterogeneous (Dover & Kelman, 2017). However, only limited empirical evidence exists that explores the dynamical aspects of this heterogeneity, how it is formed, and whether it is a predictor of network survival. Further, most empirical works consider a single network with limited longitudinal or cross-sectional coverage. To appreciate the mechanism underlying centrality heterogeneity, in this study, we leverage data consisting of more than 10,000 network online communities spanning over a decade of activity. These communities can be represented as networks by considering the users as nodes and the communication interactions as links. Interestingly, we find that inequality in the centrality of the nodes emerges with increasing network size. The average inequality of in-links is negligible when the network is small, but grows sharply with size. Evidence shows that this occurs in congruence with the emergence of a specific and universal type of network structure. Our data show that the centrality heterogeneity can be characterized largely as a division of the network structure to a nucleus of nodes and a sparsely connected periphery. A typical networked online community is composed of a relatively small nucleus “surrounded” by a large number of peripheral actors. We characterize the nucleus and the periphery based on link density and find that this structure is prevalent across all networks in the data. Further, we find that the nucleus part of the network is almost impenetrable and long-lived, and is characterized by high centrality and highly active actors. The peripheral “cloud” of actors is larger than the nucleus in its size, but is characterized by low activity and high user turnover. Namely, it is short-lived. Notably, we also find that the size ratio of the nucleus to periphery predicts network survival and that networks are stable within a narrow range of nucleus-to-periphery size ratios. Stability, here, is operated as the tendency to maintain size and network activity. When comparing to a generalized preferential attachment process (Barabasi & Albert, 1999; de Solla Price, 1986), we find that the dynamics might be similar (but not identical) with those of a preferential attachment process in the medium and large groups, but very different in the small ones. This suggests that in-links dynamics of smaller networks is determined by a more egalitarian process in which actors reciprocate links. In larger networks, the underlying heuristic that actors use to choose their network links may be popularity-based, rather than based on local and more intimate considerations. Finally, to gain better insight into the dynamic processes that shape the degree centrality distribution, we look at the within-network *social mobility* and other aspects of the network nucleation process. Social mobility, in our context, is the tendency of actors to change their network degree. We focus on how members fluctuate between centralities and whether the network has a steady-state universal structure. In what follows, we further discuss the relationship of social mobility and the nucleus-to-periphery structure to existing literature. In the rest of the paper, we describe the data and then the results. We conclude and discuss suggestions for future research in the discussion section.

### Social mobility and nucleus-to-periphery structure

Social mobility, i.e., the tendency of actors to change their connectivity over time, is potentially an important construct in actual networks (e.g., see the seminal models in (Bonabeau et al., 1995; Stauffer, 2003)), as network links can be costly and hard to maintain over time. Thus, a fundamental question is: What kind of mobility patterns should we expect in networks that are stable versus networks that are not? Furthermore, the following question is still open: how is network stability associated with certain configurations of centrality inequality, i.e., the manner in which popularity is distributed across the network (Fan et al., 2017)? Is it best that all actors are equally popular, or is it important that a small nucleus of actors “commands” the majority of the links? Most likely, the answer lies somewhere in between these two scenarios. The sociological literature suggests, mostly by theory, that the role of a densely connected nucleus is to provide the foundation for the network activity and to essentially glue the network together (Oliver et al., 1985; Oliver & Marwell, 2001). On the other hand, the role of the periphery is less clear. One theory alluded to in the literature is that the role of the actors in the fringes of the network is to interact with the nucleus, act as a channel for innovations from external sources, and trigger the activity in the nucleus (Granovetter, 1973) either by interaction, or by simply serving as an audience to it. If this is true, then we would expect the size of the periphery to be an important variable that correlates with network longevity. In other words, we hypothesize that there should be a balance between the sizes of the nucleus and periphery. A nucleus with little or no periphery may not remain active for long. In contrast, a network with a nucleus that is too small to support a large periphery population will also be less than optimal for network longevity. Here, we explore whether this point of balance between the two phases exists using real-life data. The role of social mobility in this context, and its relationship with stability,^2^ is also explored. Finally, in the literature, network temporal analysis usually focuses on a network time slice on which structural approaches are used. Notable examples are the k-core approach (Carmi et al., 2006) and community detection algorithms (Newman & Girvan, 2004). Here, the high temporal resolution of the data affords a more dynamic approach to measure social mobility within the network. To gain insight into social mobility dynamics, we estimate the chances of a newly-joined actor to climb up the social ladder and remain there. By zooming in on the dynamics within networks, we hope to uncover the origins of the heterogeneity and inequality of network centrality.

## Data

To find answers to the above questions, we use a rich longitudinal dataset of 10,122 online discussion communities with 134,747 users, using a total of 9,986,206 posts across more than 12 years. We collected the data from Tapuz forums (www.tapuz.co.il/forums), a website that allows users to form and control their own discussion communities. This setup allows us to observe community time lines in minute-to-minute resolution. For our purposes, we define a social connection between user *i* and user *j* if they interacted within a community at least once. Fortunately, this website indicates when users respond to specific messages from specific users. The networks we observe are therefore networks of active interaction, and so it is possible to estimate their real-time centralities per each time period. We describe our findings in what follows.

## Results

### Inequality grows with network size

**Fig. 1:**
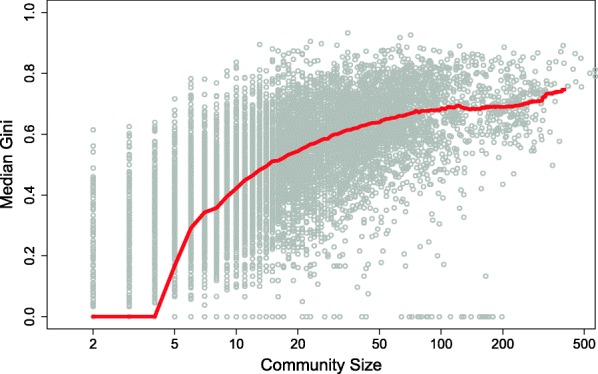
The median Gini coefficient of online communities degree centrality as a function of the size of the community. Community lifetime was divided into 60 days windows. Size and Gini were calculated for each time window separately

Figure 1 presents a visualization of the relationship between the Gini inequality coefficient (Atkinson, 1970) of user degree and community size. The lifetime of each community was divided into time windows of 60 days. Each scatter point in Fig. 1 is the result of the measurement of the Gini coefficient vs. network size within each window, for each community. The solid red curve represents the median Gini coefficient as a function of community size within a time window. The median was preferred over the mean for representing the distribution of Gini coefficients because it is less sensitive to outlier values. We also chose to use the Gini coefficient as a measure of centrality inequality and, specifically, the inequality of user in-degree. In-degree in online communities effectively measures social attention or popularity, i.e., the time and effort that an actor attracts from their peers. Fig. 1 presents hints towards the evolution of a centrality inequality, i.e., how it grows with growing network size. The median Gini coefficient across size-bins initially is flat, up until communities of four individuals and then, starting at groups of four actors, it monotonously increases until it flattens again for large groups. It seems that as communities grow, they naturally also become more and more unequal in terms of peer-to-peer attention and up to a plateau of a Gini coefficient of 0.8. To get an idea of the level of inequality in the figure, Gini levels in world economies: the economy of Botswana tops the inequality list with a Gini of 0.63 (South Africa in 0.62), while Sweden has an estimated Gini value among the lowest, of 0.25.^3^

These findings suggest that there is, presumably, a difference in the formation of small networks and larger networks. The formation process of small networks (up to about 5 members) results in a relative uniform distribution of connections, while for larger networks, connectivity inequality grows strongly with growing network size. In fact, it is this small densely-connected seed group^4^ that, later as the network grows, comprises the nucleus. Newcomers receive less and less in-link attention; thus, a sizable gap forms between the popularity of the original early small nucleus of users and the (much larger population of) newcomers.

An alternative explanation is that these patterns are merely due to an age effect, i.e., even though newcomers start with a popularity disadvantage, they will accumulate popularity over time, replacing older members and enabling a positive turnover rate in the nucleus. However, this is not what we find in the data. As discussed in section “Transient populations in outer shells nucleate around a highly stable nucleus”, turnover in the nucleus is negligibly small relative to the visible turnover rates of the periphery. Unlike models that predict the formation of online networks by a process of coagulation around several communities/nuclei, we find that online discussion forums nucleate around one major central core, thus leading to a high level of popularity inequality.

**Fig. 2:**
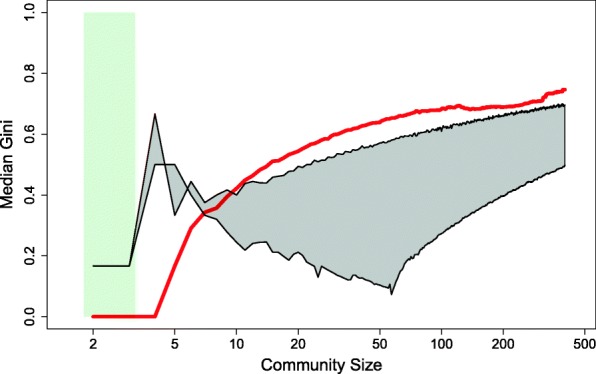
Measure of degree inequality within the community plotted against size. Red is the median line of networks from real-life data, the grey envelope are simulations of Generalized Barabasi-Albert networks with 0.1 ≤ *α* ≤ 2, and the light green band marks the range where we used the ordinary Barabasi-Albert

In order to qualitatively compare our observations to one of the leading models of structure formation, we simulated a wide range of Gini-vs.-size curves in the Generalized Barabasi-Albert model (Pham et al., 2015) and overlaid the results on the empirical Gini-vs.-size curve, as seen in Fig. 2. The Generalized Barabasi-Albert model attachment function is of the form *A*_*k*_ ∝ *k*^*a*^ where *α* > 0. The envelope depicted in the figure, marked by the gray area and bordered by black curves, covers a wide range of alpha values in the Generalized Barabasi-Albert model. Specifically, this envelope spans the simulated results of the Gini-vs.-size median curve of *α* = 0.1 (top curve) and the curve of *α* = 2. We deployed 500 instances of simulated Generalized Barabasi-Albert networks per each community size in the range of 3 ≤ *N* ≤ 400. The simulations were split to two parts: networks greater than three members were simulated with the general algorithm because three is the minimal number that the Generalized Barabasi-Albert algorithm allows. Smaller community sizes were generated using the ordinary Barabasi-Albert model (Csardi & Nepusz, 2006). The range of sizes which were generated by the ordinary Barabasi-Albert model are depicted by the light green band in the figure. In Fig. 2 two patterns stand out: (1) small communities are highly equal, on average, especially relative to the preferential attachment models and: (2) real-life inequality exceeds the highest preferential attachment levels of inequality across two orders of magnitude of network sizes. In that sense, it seems that the data suggest that there are two regimes of dynamics that govern networks, according to size. When the network is small, at the stage in which its nucleus forms, the dynamics are more-egalitarian, i.e., reciprocal. But, when the network grows in size and expands, these reciprocal dynamics do not translate into the rest of the network. The nucleus retains its high level of connectivity and accumulates more popularity with increasing size, while the periphery does not; thus, high levels of inequality emerge. In the next section, we zoom in on the social mobility within a network to explore if, indeed, the patterns that we hypothesize, govern turnover within network communities.

### Social mobility is negative on average

**Fig. 3:**
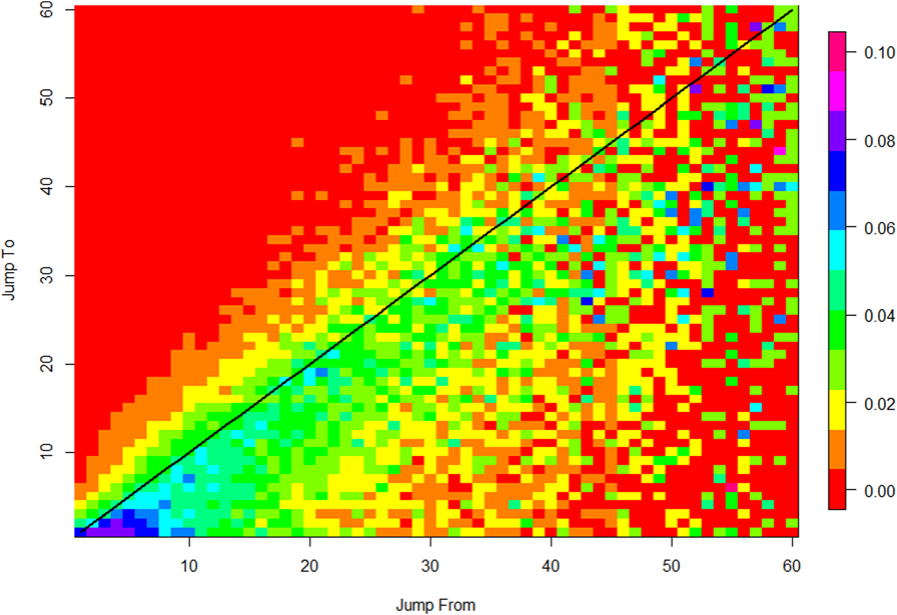
A heat map of the degree transitions over time. The X-axis marks the degree in the current period and the Y-axis marks the degree in the following 60 days. The probability of a transition from degree X to degree Y is color coded

Figure 3 shows a transition matrix where we color-code the probability of a member to shift from degree *i* to any other degree *j*, during a time period within the community. A guiding black line marks the stable case *i* = *j*. The time window used for calculating the matrix in Fig. 3 is 60 days.^5^ For example, the color of the point {10, 20} in the figure, illustrates the probability that actors who had 10 connections in the previous 60 days period, will have 20 connections in the 60 days period that follows. The estimations were done across communities that exhibited activity allowing meaningful interaction.^6^ The figure shows a strong negative drift across degree, i.e., it is much harder to climb up the centrality “ladder” than it is to drop down. The figure also suggests that degree tier turnover is the greatest for degrees k < 20 and drops beyond this value. The second observation is that the expected dynamics seem somewhat different for degrees above and below k = 40. For k < 40, there is a dense concentration of points in which there is a narrow range of high probability that is exhibited by a green stripe that stretches along and below the *i* = *j* line. Our interpretation of this is that, while the data show that transitions exist above and below k = 40, most of the time, communities exist in the lower tier of degrees and that there is difference between the low- and high-degree regimes. For the k > 40 regime, there are also high probability clusters (seen as blue points) below the solid reference line which mark more-probable jumps to lower degrees, i.e., negative social mobility. This suggests that the high-degree region is more unstable.

### Most nodes do not make it into the nucleus

**Fig. 4:**
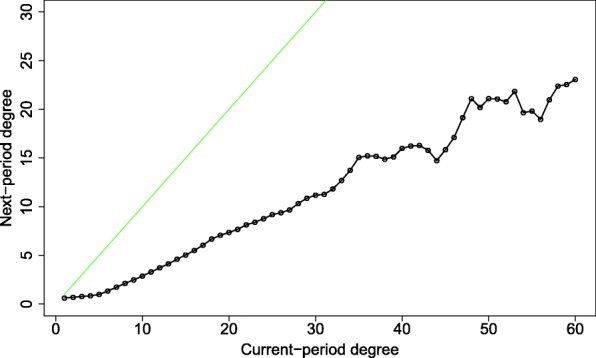
The expected next-period degree (Y-axis) as a function of the current-period degree (X-axis) is marked by the black dotted line. The next-period expected degree was calculated per current-period degree using the respective column in the transition matrix (Fig. 3). The green line marks the stability reference - when the expected next-period degree is equal to the current-period degree

**Fig. 5:**
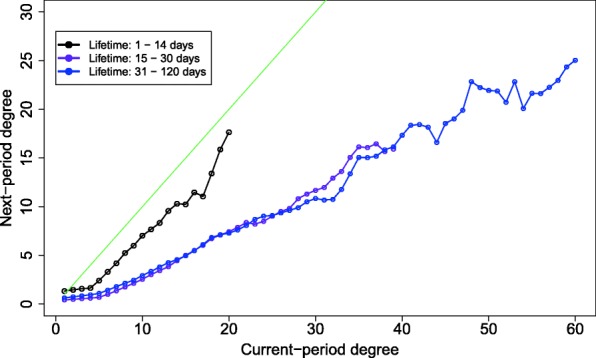
The expected next-period degree (Y) vs. the current-period degree (X) for three stages of actors’ lifetimes: less than two weeks, two weeks to a month, and one to four months. Curves were calculated as in Fig. 4. The stability reference line is in green

Figure 4 illustrates the curve of the expected degree (black) in the next time period, per each degree *i* in the current time. The figure clearly shows that, overall, social mobility is negative, consistent with previous findings, i.e., that the next-period degree’s expectation line lies below the stable community line (here in green). On average, individuals constantly lose connections. The instability increases with growing degree, as is seen by the growing gap between the expectation and stability lines. The general picture in Fig. 4 is that the influx of actors into the high-centrality nucleus is low. An alternative explanation for the patterns we observe is that of activity fatigue, or, an expected activity life cycle. In this scenario, the observed patterns are the result of a natural decay of the average user’s activity levels. Initially, a typical actor will be active and climb up the ranks. Then, after a certain period, activity will slow down, and the actor will gradually lose in-links. First, this alternate story is inconsistent with the observed high stability of the nucleus (see section “Transient populations in outer shells nucleate around a highly stable nucleus”). Second, in this scenario, we would expect newcomers to exhibit low degrees and positive social mobility, while negative social mobility will be common among more senior actors. To explore whether it is true that newly-joined actors exhibit positive social mobility, Fig. 5 gives the social mobility for different durations of activity (actor activity “ages”). The patterns in the figure do not support the activity fatigue effect as an alternative story. The figure shows that Short-lived actors, who were active for less than 2 weeks (solid black), accumulate degrees up to k = 20. This is expected since we assume that gaining connections takes time.

Interestingly, very young communities (up to 2 weeks old), exhibit higher social mobility, relative to the older communities we explored. Although this is out of scope of the paper, it could be a sign that communities undergo an initial period of stabilization of social mobility, starting with higher rates and later settling on lower levels, as is seen for older communities. But, it is clear that even at this very early stage, communities exhibit negative mobility, regardless of age and a low influx of actors into the nucleus.

### Transient populations in outer shells nucleate around a highly stable nucleus

**Fig. 6:**
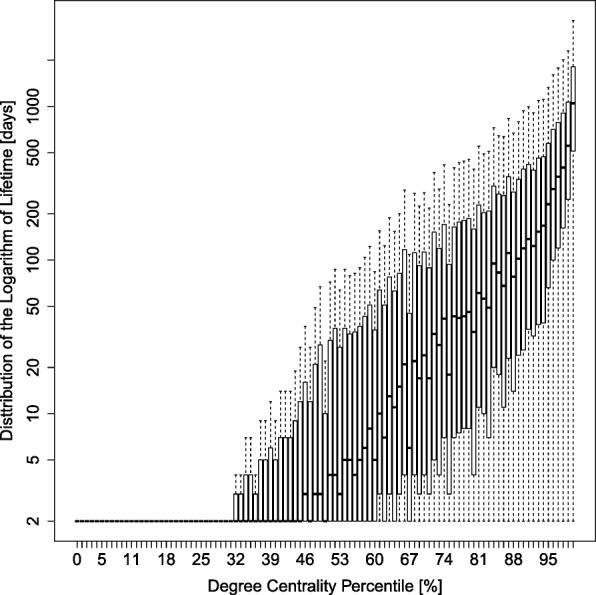
Persistence in the community. A box-and-whisker plot of the distribution of user activity log-lifetime (Y-axis) per degree grouping X. The base of the log function is *e* here. Each equal-count centrality group is 1 %. The median lifetime is represented by thick black marks

The box plot in Fig. 6 shows the distribution of the logarithm of activity lifetimes (in days) for each centrality percentile, calculated across all 10,122 communities. In order to compare between online communities of varying sizes, we consider lifetime vs. centrality percentiles. Within each community, we partition the users to 101 equal-count groups according to degree, whereby each user is assigned a discrete position from 0 to 100. For example, 90% on the X-axis denotes individuals with degree-centrality greater than 90% of the degrees within their community, but lower than 9% of them. Per each actor, their lifetime within the community is calculated by their assigned centrality percentile score. The thick horizontal line, per percentile, marks the median lifetime across all actors of a given percentile score. The 2nd and 3rd quartiles are denoted by a box around the median, and the dashed lines denote the 1st and 4th quartiles of the distribution. Figure 6 clearly shows a strong divide between parts of the network that have varying degree centralities. Up to around the 48th percentile, the median lifetime is very short. Above this percentile, lifetime increases non-linearly with percentile. For example, the lifetime of the 98th percentile is three orders of magnitude higher than that of the members in 50th percentile. This, along with our findings regarding social mobility, provides the following insight into the centrality dynamics. The network is composed of subnetworks of varying connectivity. The more connected a sub-network is, the more stable and long-lasting it is. Given that the social mobility is largely negative and drops significantly for higher degrees, we can conclude the following ≠ it is hard for actors to climb up and join the high-connectivity nucleus, but once an actor penetrates it (or is already inside), the actor stays there for long periods. The outer shells, on the other hand, constantly turn over and are short-lived. These dynamics seem to be prevalent across more than 10,000 communities of varying topics and populations, and for more than a decade. In light of the stable two-mode (nucleus-to-periphery) structure across communities, the following questions are still unanswered: To what extent is it related to the stability of the network? What is the optimal size of a nucleus, versus periphery for a network to survive? Small or large? Is the short-lived and less connected population redundant, or is it critical to the longevity and activity of the network? In what follows, we explore how social mobility and certain compositions of nucleus and outer shells predict community growth, or demise.

### Low social mobility is associated with lower network longevity

**Fig. 7:**
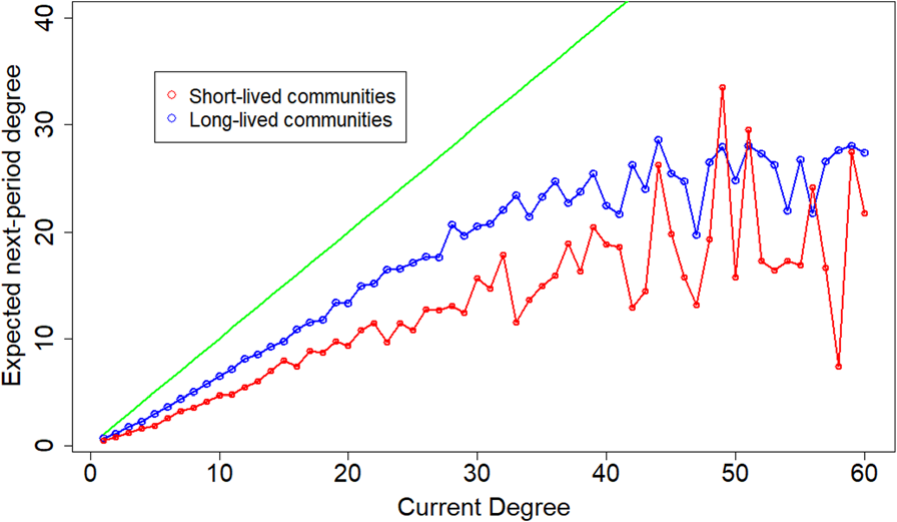
Similar to Fig. 4, the expected next-period degree (Y-axis) is plotted against the current-period degree (X-axis). The blue line marks the long-lived communities and the red line marks the short-lived communities. The solid green line is the stability scenario X = Y

**Fig. 8:**
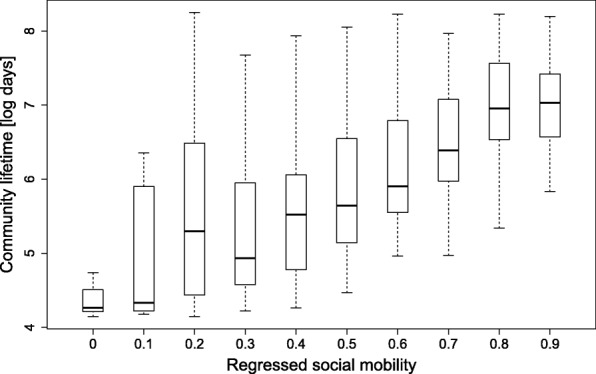
A box plot of the distribution of the natural log of community lifetimes in days, as a function of regressed social mobility. The solid horizontal line in each box represents the median and the box represents the 2nd and 3rd quartiles. The dotted lines designate the 1st and 4th quartiles of the lifetime distribution. Only communities with more than 200 posts were used in the analysis

We divided all communities in the data into two groups, short- and long living, using median community lifetime as the cutoff threshold. Community lifetime is the time from the first post within the community of any actor until the time when the last community activity is recorded. We define the short-lived (long-lived) communities as those that have lifetimes of less or equal to (greater than) the overall median lifetime, which is 317 days. Figure 7 shows that the average social mobility is negative, and different between the two groups. The instability (shorter lifetime) of the short-lived networks (red curve) is greater such that these communities find the “social ladder” consistently harder to climb, irrespective of the typical user’s degree. Further, the degree-dependent negative drift increases at a faster rate in short-lived communities with increasing degree. Long-living communities allow more centrality turnover, especially into the higher degrees. In that sense, the long-lasting communities seem to be supportive of a more “equal opportunity” scenario when it comes to social mobility of users. A different approach to exploring the relationship between social mobility and community lifetime, is the following. For each community we estimate the magnitude of social mobility by using regression analysis on how the current degree predicts future degree, per each time window of 60 days, and per user within a community. This allowed us to compare each community’s social mobility (as expressed by the regression coefficient of current degree) to its lifetime in days. The distribution of community lifetimes as a function of the regressed social mobility is shown in the form of a box plot in Fig. 8. The figure shows, consistent with our other findings, that higher social mobility is associated with longer community lifetimes. In sum, we observe that social mobility is negative and that higher mobility is associated with higher stability. Also, as discussed above, we observe that it is almost impossible to penetrate a highly-connected nucleus.

### Network survival is associated with a unique proportion of nucleus-to-periphery size

**Fig. 9:**
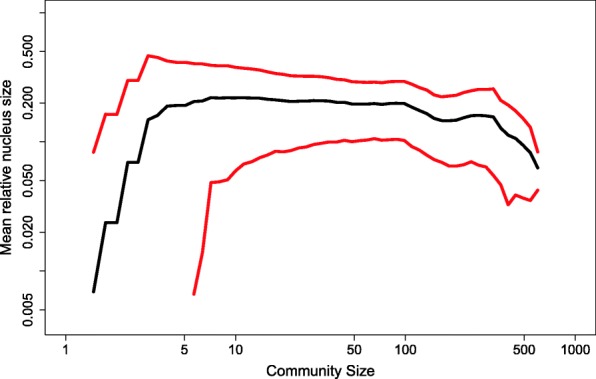
The average nucleus-to-periphery size ratio is denoted by the black curve. The red curves denote one standard deviation around the average

**Fig. 10:**
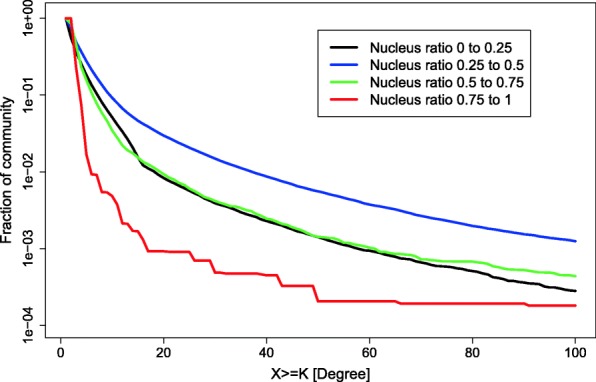
The fraction of actors in a community with a given degree K or above (X axis), for varying ranges of nucleus-to-periphery ratios (see legend)

The relationship between the nucleus-to-periphery size ratio and social network properties is relatively unexplored in the literature. The black curve in Fig. 9 illustrates the average nucleus-to-periphery ratio as a function of community size. Interestingly, it seems that for communities smaller than 5 members, the nucleus is small and under-developed. This is consistent with the fact that very small communities exhibit relative equal connectivity among their members, and that inequality (in the sense that there is a separation between dense nucleus and sparse periphery) develops only when communities grow larger. For larger communities, the ratio is mostly around 0.20 but mildly declines with size. For very large communities, of above several hundreds of members, there is steeper decline. The relationship between degree centrality heterogeneity and the relative size of the nucleus is demonstrated in Fig. 10. Each curve corresponds to a group of communities in a certain range of *nucleus-to-periphery ratios*. The nucleus-to-periphery ratio, *η*, is the proportion of actors that form the nucleus over the size of the whole community. The blue curve, calculated for communities with ratios in the range 0.25 ≤ *η* ≤ 0.5, shows the highest heterogeneity of degree. For some reason, high relative connectivity develops in communities in this range. In ranges that are lower or higher than those denoted by the blue curve, the fraction of members drops faster in low degrees and thus the frequency of high degrees tends to be smaller. We interpret this as suggestive that nucleus-to-periphery ratios in this range serve better basis for high-connectivity groups to emerge.

**Fig. 11:**
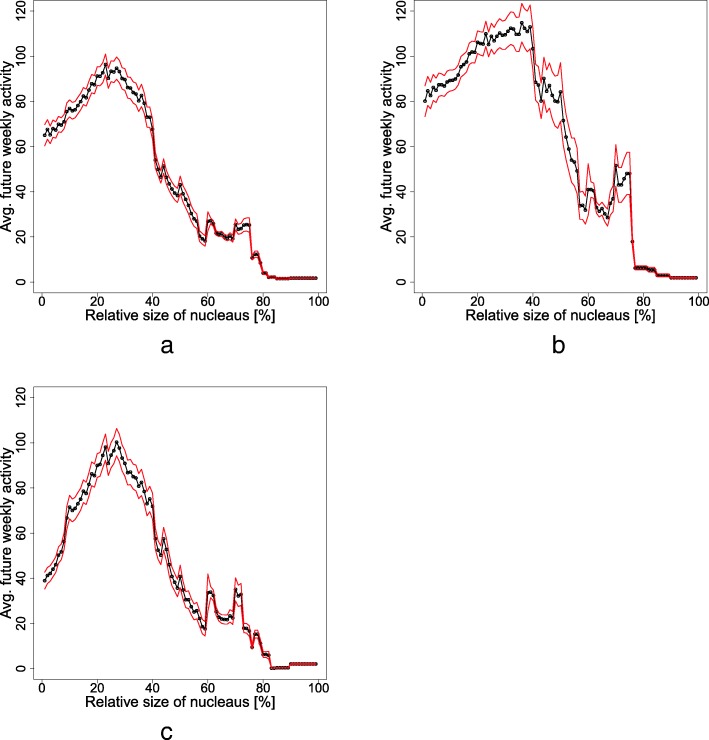
Future activity $\widehat{A}$ of a community as a function of the fraction of actors in the nucleus N. Members of the nucleus are defined to have a local clustering coefficient of 1 and are identified on the network, per each time slice. Future activity is the total of messages posted during a 60-day time frame following the identification of nucleus members. Mean future activity per nucleus size is shown in black (standard error in red). **a** The calculation across all 10,122 communities. **b** The subset of long-lived communities (above-median lifetime). **c** The short lived communities (below median lifetime)

In the context of these last observations, it is of interest to try and test whether nucleus-to-periphery ratios can predict future community activity. Figure 11 is an illustration of how the ratio of nucleus-to-periphery size is a predictor of future community activity. The X-axis marks the nucleus-to periphery ratio, *η*. We choose a definition that allows a clear and discrete distinction between nucleus and periphery: an actor is part of the nucleus if their node is embedded in a fully connected neighborhood, or formally, with a clustering coefficient of unity. In each 60-day time slice, we construct the interaction network between actors and estimate the local clustering coefficients. Then, we classify whether users are members of the nucleus or not, using their local clustering coefficients. Last, we calculate *η*for that community during that time slice. We plot the average weekly activity $\widehat{A}$ community’s next frame as a function of the proportion *η* (X-axis). Future activity is the total activity of all actors in the following time slice (Y-axis). We generate *η* and $\widehat{A}$ curves in the figure denote the sampling standard error. Notably, Panel 11a shows that there is a narrow nucleus-to-periphery ratio that predicts maximal activity across all communities. Interestingly, the peak lies at around 25% nucleation, i.e., a 1:3 ratio between nucleus size and the periphery. It is also clear from the figure that as a network departs from that exact ratio, the predicted future activity diminishes. We interpret the patterns in Panel 11a as suggesting that networks overwhelmingly dominated by their nucleus do not survive for long. This can be seen by the rapid diminishing of future activity for high nucleus-to-periphery ratios. Conversely, to survive, a network requires a relatively large population in the outer shells. The theoretical literature stresses that the nucleus is responsible for the better part of the network activity (Oliver et al., 1985). While our findings are consistent with that, here, we show that focusing only on the high centrality nucleus is not enough to gain useful insight into network stability. The relationship between the nucleus and periphery is a better indicator and seems to be a crucial variable that predicts stability. In fact, Panel 11a shows that there is a certain relative nucleus-to-periphery size ratio that is a predictor of stability and survival. Consistent with the literature, we interpret the effect of the external short-lived periphery to be an effect of “fertilization” within the network. The large periphery enriches the nucleus and the network with new ideas, external opportunities, and innovation (Granovetter, 1973). A major observation that one can extract from Panel 11a is that there exists an optimal proportion of nucleus-to-periphery sizes. Using our interpretation, it is possible to hypothesize that within the optimal range of nucleation, a balance is reached between the need of the network to have a large enough nucleus that dominates activity vis-a-vis the need for a large mass of peripheral population that will enrich and trigger the nucleus population.

To gain more insight into the optimal nucleus-to-periphery ratio, we repeated the analysis for short- and long-lived communities, again using median community lifetime as the determining cutoff. The results for the short-lived communities are illustrated in Panel 11c. This panel shows that the unique nucleation value of about 1:3 is crucial to younger communities. Deviation from the ratio translates into a collapse of network activity. Long-lived communities, on the other hand, show more flexibility, in that they can host a wider range of nucleus-to-periphery ratios, as seen in Panel 11b. We interpret this as evidence that older communities that have already established patterns of social interaction could support both larger and smaller nuclei. Interestingly, the ratio in which future activity peaks has a wider range for old communities, suggesting that older communities thrive with relatively large nuclei. The reason for this might be either because these well-established communities require less peripheral support, or that they can host larger nuclei.

A more detailed exploration of the role of community age in the relationship between nucleus-to-periphery ratio and future activity, is illustrated in Additional file 1: Figure S1. In that figure, we divide communities into six tiers of age. The situation depicted in that figure is a bit more complex. As communities age, the optimal nucleus-to-periphery ratio spreads out and then for communities of 2 years and older, it narrows again at a lower value. In other words, as communities reach very old ages, a small nucleus is associated with more activity (even though, in general, future activity wanes as communities mature).

## Discussion

In this paper, we present data of online communities where the actors and their interactions inside a community can be represented as a network. We study the relationship between the network’s size and the heterogeneity of its degree centrality. We also explore the relationship between dynamical stability and social mobility, whereby in each community a high-centrality nucleus, and sparser outer shells, exist. We term this proportion the *nucleus-to-periphery ratio*,*η*. In general, we find that the network is composed of two sub-structures that correspond with two dynamical patterns, or phases: the large and highly mobile “cloud” of loosely connected users that nucleates around a practically impenetrable nucleus of tightly connected members. From real-life data, we find evidence that the nucleus-to-periphery configuration of a network is crucial for its stability and survival. Remarkably, a specific narrow range of the nucleus-vs-periphery ratio, $\eta \approx \frac{1}{4}$, predicts network success across more than 10,000 communities, over more than a decade of activity. This is especially true for younger networks.

Even though our findings do not offer complete causal validity, we argue that they do supply fundamental insight. Notably, one contribution of this work is to emphasize the significance of the myriad but less popular users whose role in achieving long-standing dynamical stability is, by and large, ignored in the literature. Our interpretation of the findings is that these actors play the role of (1) triggering the nucleus users by being the “audience”, and (2) enriching the interactions in the community by bringing in external information and ideas. A direction for future research is to further investigate the role of network actors in affecting network function and stability, on both the micro and macro levels. Possibly, research should explore the dynamics of the varying lifetime stages of networks using data similar to ours. A more complete body of research of the relationship between network structure and stability could, perhaps, enable the engineering and fostering of long-lasting and stable social groups.

## Additional file

Additional file 1: **Figure S1.** The relationship between current-period nucleus-to-periphery size ratio and future weekly activity, for varying community age tiers. Nucleus-to-periphery ratio were calculated for each community, within time windows of 60 days. Future weekly activity was calculated, per each time window and correlated with the previous 60 days time window. (PDF 17 kb)

## Footnotes

1 In this work, we focus on degree centrality, i.e., the number of links that an actor maintains in a social network.

2 In general, we measure stability by looking at a community’s tendency to remain active or maintain and/or attract members.

3 OECD statistics, Social Protection and Well-being ≠ Income Distribution and Poverty - by country - INEQUALITY. OECD. 2016. http://stats.oecd.org

4 It is noteworthy that although this scenario seems similar to that of preferential attachment, we do not claim that preferential attachment is the underlying scenario. Any scenario in which the seed group becomes the nucleus in a larger older network, will be consistent with our findings.

5 Varying the lengths of the time windows keeps the results qualitatively similar. The 60-day time frame was eventually chosen because it is large enough to register sufficient activity, while still allowing a sizable set of temporal observations

6 We selected communities with 200 posts at the very least, a total of 1,127 communities.
