# Supplementary figures and images for: Centralized nucleation in online networks leads to high social inequality
Source: Appl Netw Sci. 2018 Oct 4;3(1):43. doi: 10.1007/s41109-018-0102-3 (PMC6214323; doi:10.1007/s41109-018-0102-3)

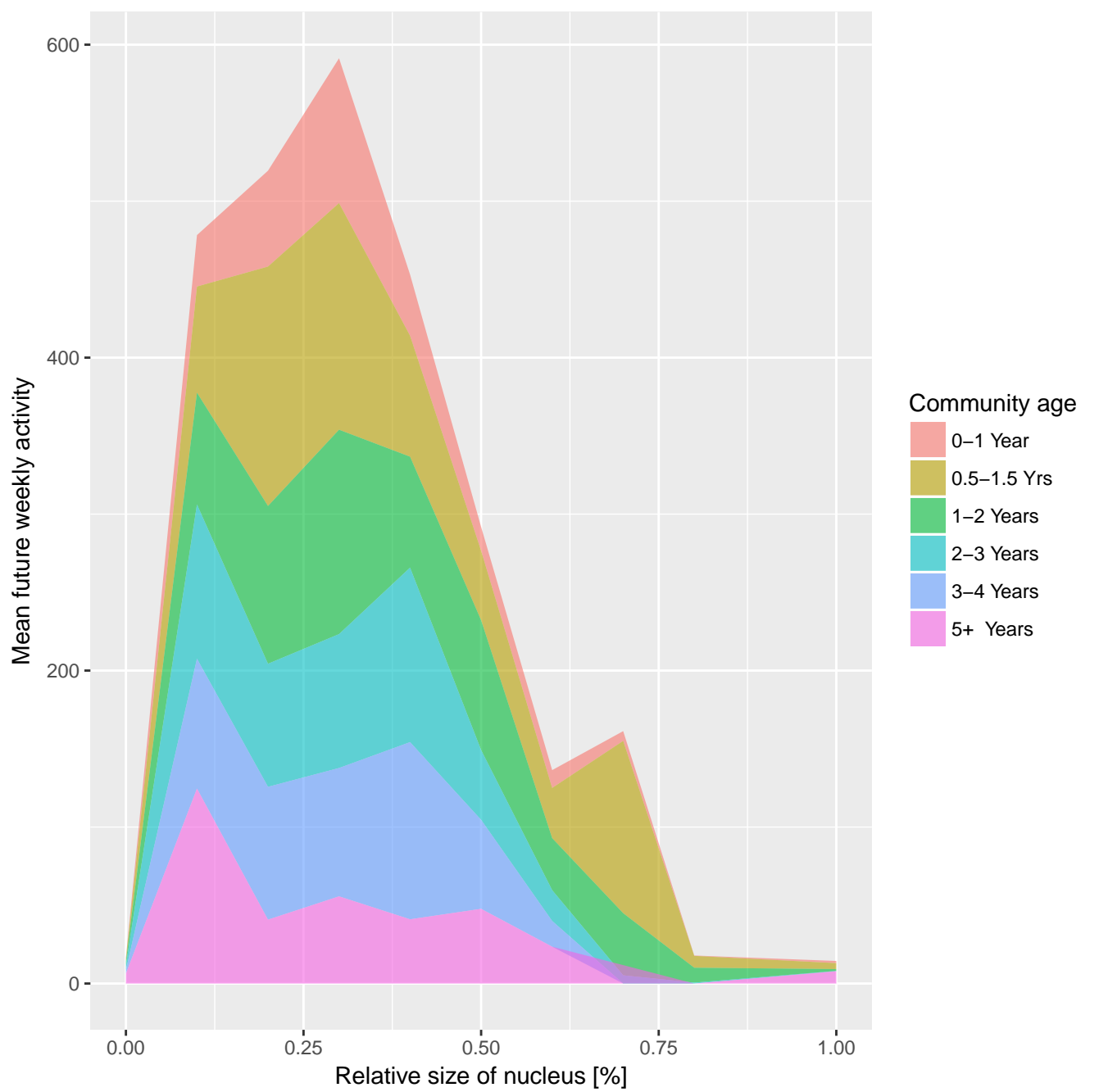

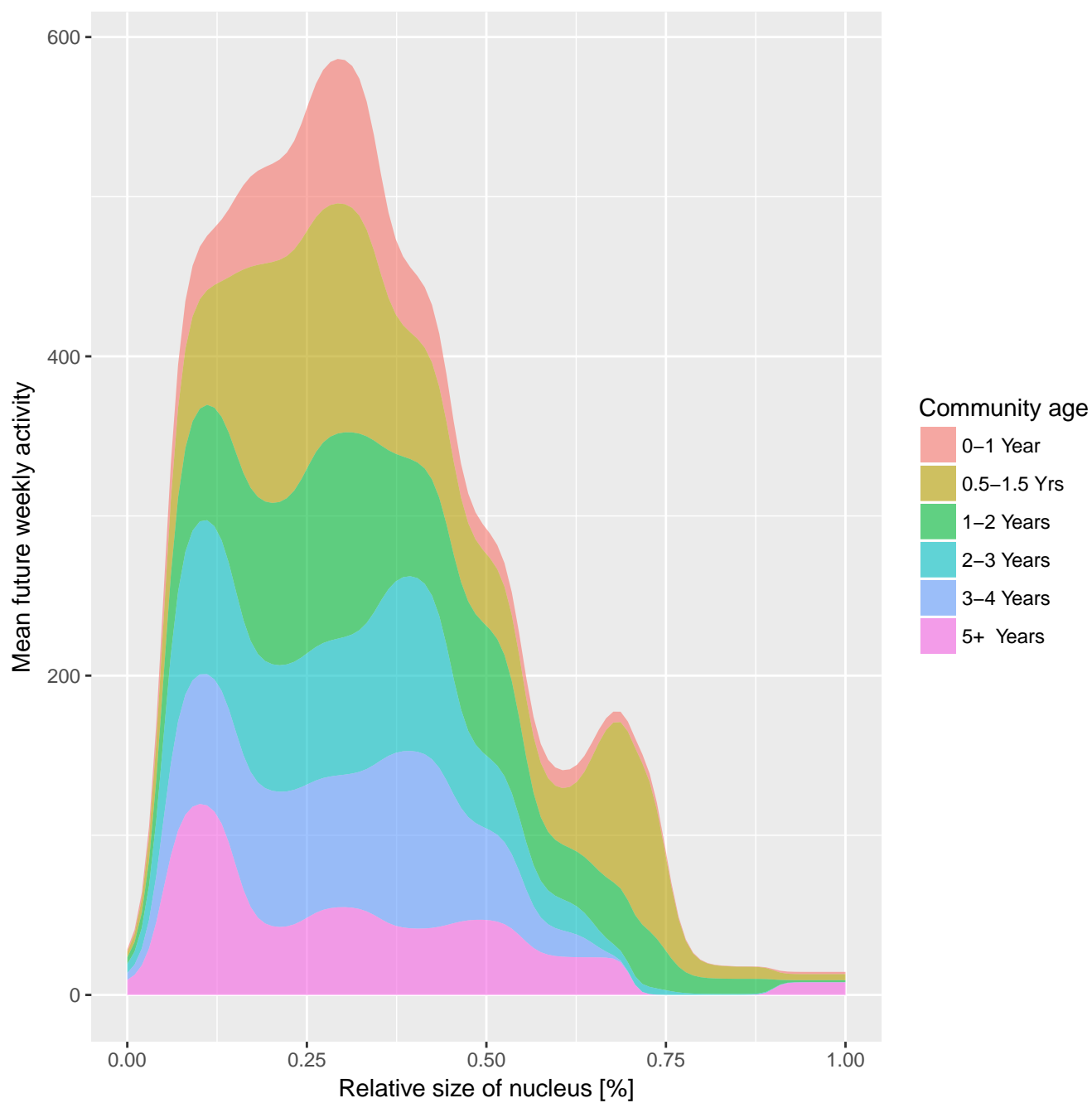

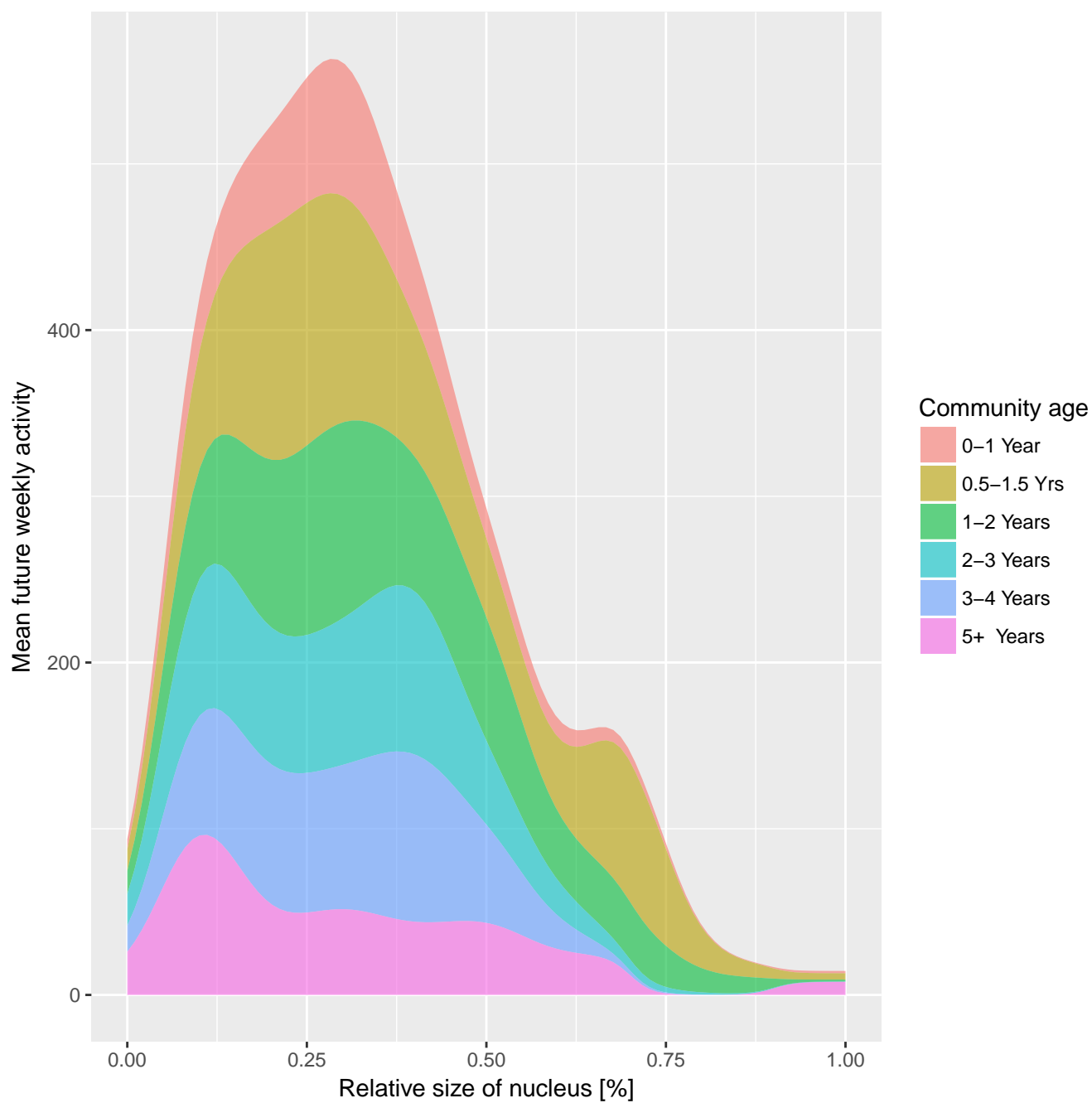

Supplement: Supplementary file 1 — Figure S1. The relationship between current-period nucleus-to-periphery size ratio and future weekly activity, for varying community age tiers. Nucleus-to-periphery ratio were calculated for each community, within time windows of 60 days. Future weekly activity was calculated, per each time window and correlated with the previous 60 days time window. (PDF 17 kb) [file 41109_2018_102_MOESM1_ESM.pdf]
